# The Cation Effect on the Free Volume and the Solubility of H_2_S and CO_2_ in Ionic Liquids Based on Bis(2-Ethylhexyl) Sulfosuccinate Anion

**DOI:** 10.3390/membranes13020238

**Published:** 2023-02-16

**Authors:** Tatyana S. Sazanova, Alsu I. Akhmetshina, Anton N. Petukhov, Andrey V. Vorotyntsev, Sergey S. Suvorov, Alexandra V. Barysheva, Amal Mechergui, Alexander V. Nyuchev, Olga V. Kazarina, Anna N. Stepakova, Maria E. Atlaskina, Artem A. Atlaskin, Sergey S. Kryuchkov, Ilya V. Vorotyntsev

**Affiliations:** 1 Laboratory of SMART Polymeric Materials and Technologies, Mendeleev University of Chemical Technology, 9 Miusskaya Sq., 125047 Moscow, Russia; 2 Department of Technologies for the Processing of Polymers and Composite Materials, Kazan National Research Technological University, 68 Karl Marx Str., 420015 Kazan, Russia; 3 Chemical Engineering Laboratory, Research Institute for Chemistry, N.I. Lobachevsky State University of Nizhny Novgorod, 23 Gagarin Avenue, 603950 Nizhny Novgorod, Russia

**Keywords:** dioxide carbon, hydrogen sulfide, ionic liquids, solubility

## Abstract

Herein, we report for the first time a study dedicated to acidic gases’ solubility in ionic liquids with sterically hindered bulky anion, namely bis(2-ethylhexyl) sulfosuccinate ([doc]), experimentally evaluated at low pressures. The effect of cation change (imidazolium, pyridinium, and pyrrolidinium) on the thermophysical properties and sorption capacities was also discussed. The densities and the activation energies of the tested ILs exhibited minor differences. Furthermore, the COSMO-RS model was used to predict the free volumes of ILs aiming to investigate its influence on gas solubilities. The conducted calculations have revealed an antibate correlation between the fractional free volume (FFV) and Henry’s law constant. In particular, the lowest FFV in 1-methylimidazolium [doc] corresponded to the minimal sorption and vice versa. In addition, it was shown that the presence of protic cation results in a significant reduction in CO_2_ and H_2_S solubilities. In general, the solubility measurement results of the synthesized ILs have shown their superiority compared to fluorinated ILs based on the physical absorption mechanism.

## 1. Introduction

Current environmental problems include the removal of acid gases, such as hydrogen sulfide and carbon dioxide. Increased attention to them is associated with the greenhouse effect caused by them, which leads to irreversible climate change, as well as a negative impact on the biosphere. Today, for acid gas removal, the industry uses a technology based on the chemisorption with an alkanolamine aqueous solution. This technology has some disadvantages, such as the high energy demand for liquid reuse, as well as solvent loss and degradation [1]. Regarding this, there have been in recent years increasing interest in acid gas separation using membrane technology, which is more economically viable and energy efficient [2,3,4]. The class of membranes, represented by ionic liquids (ILs) or their polymeric derivatives, revealed excellent promises toward acid gas separation characteristics, and the ratio of ideal selectivity to permeability were above the Robeson upper-bound [5,6,7,8].

Several studies have shown the remarkable solubility of polar gases into ILs via physical dissolution [9]. Among them, the highest absorption capacity was observed in the ILs containing highly fluorinated anions. To simulate the interaction between gases and ILs, researchers successfully applied the methods of molecular dynamics simulations [10,11,12], Cosmo-RS [13], and other calculation methods. Zhang et al., using the COSMO-RS method, proved that the longer the fluoroalkyl chain in the anion (e.g., [FAP] anion), the higher the CO_2_ solubility [14]. However, fluorinated anions commonly tend to hydrolyze under humid conditions and release in the environment aggressive hydrofluoric acid [15]. On the other hand, the chemical interaction of acidic gases with the functional groups are present in the structure of the ionic liquid, particularly amine groups, which represent an alternative strategy for the effective removal of such gases. Nevertheless, the CO_2_ capture in amine-tethered task-specific ILs is generally accompanied by the dramatic increase in the absorbent’s viscosity due to the formation of strong hydrogen-bonded networks between the zwitterion and dication species [16]. Another strategy of CO_2_ chemical absorption is focusing on the yielding of adducts with carbene species acting as anions, for example imidazolate, benztrazolate, and pyrrazolate anions [17], or being a part of cations, as in the case of 1-butyl-3-methylimidazolium acetate [18]. Although the latter type of absorbents is characterized by the outstanding sorption properties toward the acidic gases, the presence of reactive carbenes may have a harmful effect on the human health [19]. Therefore, the application of ionic liquids binding the acidic gases via the chemical dissolution has limitations augmented additionally by the difficult desorption and high energy demand for regeneration.

According to the above, for effective acid gas removal, the focus should be on the new environmentally friendly ionic liquids (ILs) creation. One of the promising approaches is the flexible fragment’s introduction in combination with polar functional groups. In particular, ether-functionalized anions permit dipole–quadrupole cooperations between the oxygen atom of the functional group and carbon atom in CO_2_, and lead to the increase in the free volume, thus raising ILs affinity toward CO_2_ [20]. Zhang et al. investigated an ionic liquid comprising 1,1,3,3-tetramethyl-guanidinium and lactate anion, which showed successful CO_2_ absorption [21]. Deng et al. [22] found that the CO2 solubility in ester- and ether-functionalized imidazolium ILs was of the same order of capacity as in alkylimidazolium ILs, and the additional functionality has a minimum impact on the solvation capability of ILs. Further molecular simulation [23] showed that the insertion of ester groups into cations can change the polar and nonpolar domains’ relative size in ILs, but has no significant effect on the gas’s solvation (such as CO_2_).

Note that in the Mortazavi-Manesh [24] study, the COSMO-RS method predicted a high solubility of CO_2_ and H_2_S in ILs containing bis (2-ethylhexyl) sulfosuccinate. It has also been experimentally proven that at high pressures, the CO_2_ solubility in tetrabutylammonium bis (2-ethylhexyl) sulfosuccinate ([N_4444_][doc]) is in good agreement with the data obtained for the CO_2_ solubility obtained by fluorinated ILs hmim[eFAP] [25]. According to the authors, [doc] anion has several features that lead to good CO_2_ solubility values, namely carbonyl functionality and long-branched alkyl chains. Therefore, it is possible to produce non-fluorinated ILs with a good capacity for CO_2_. In addition, the sorption properties of ILs with an anion [doc] at low pressures have still not been investigated. In addition, because of the high viscosity of ether-functionalized ILs, the CO_2_ absorption rate may not reach a maximum. Viscosity control is achieved by reducing the number of hydrogen atoms on the anion available for hydrogen bonding, or by adding some organic solvents or water, which helps to achieve optimal CO_2_ absorption results in ILs.

Whereas detailed information on a wide range of fluorinated ILs and their sorption properties are published in various literature sources devoted to modern methods of acid gas removal, information on the effect on the acid gas’s dissolution in sterically hindered and bulky fragments together with polar functional groups, such as ester groups, have not been studied enough in the literature. This work focused on the influence of the free volume on acid gases’ solubility, especially carbon dioxide and hydrogen sulfide, in four ILs synthesized by amphiphilic bis (2-ethylhexyl) sulfosuccinate anion and imidazolium, pyridinium, and pyrrolidinium cations.

## 2. Experimental Section

### 2.1. Synthesis of Ionic Liquids

The synthesis of ILs employed in this investigation was completed by the formerly described anion exchange reaction [26]. The ILs were synthesized using the sodium bis(2-ethylhexyl) sulfosuccinate reaction (96% purity, Sigma Aldrich, Darmstadt, Germany ) and the corresponding heterocyclic salt, namely 1-methylimidazolium chloride (95% purity, Sigma Aldrich), 1-ethyl-1-methylpyrrolidinium bromide (99% purity, Sigma Aldrich), 1 butyl-3-methylimidazolium chloride (≥98% purity, Sigma Aldrich), or 1-hexylpyridinium bromide, in an acetone medium. 1-hexylpyridinium bromide was preliminary synthesized through the quaternization of pyridine (99.8% purity, Sigma Aldrich) by 1-bromohexane (98% purity, Sigma Aldrich). An anion exchange by-product, such as sodium chloride or sodium bromide, was removed by filtration. Acetone was evaporated under reduced pressure on a rotary evaporator (RV 10 digital, IKA, Staufen, Germany) with vacuum pump LVS 105 T-10 ef (IKA, Germany). The ionic liquids were washed five times with deionized water until the aqueous phase began to form a precipitate with a 0.05 mol/L AgNO_3_ solution, after which it was dried. The synthesized IL chemical structures were identified by ^1^H and ^13^C NMR spectra. All ILs were desiccated under a vacuum for 24 h, after which the water content was measured by Karl–Fischer titration (Coulometer 831 KF, Metrohm, Zofingen, Switzerland). The water content is presented in Table 1.

### 2.2. IR and NMR Spectroscopy

Spectroscopy methods (IR, ^1^H, and ^13^C NMR) were used to determine the structure and purity of the synthesized ILs. IR spectra were registered at ambient temperature on an IRAfinity-1 IR-Fourier (Shimadzu, Kyoto, Japan). A minimum of 30 scans were averaged over a signal with a resolution of 4 cm^−1^ at the 2000–800 cm^−1^ range. The remaining parameters were not controlled and correlated with the specifications revealed by the manufacturer. Wave number measurement accuracy was controlled by the polystyrene spectrum, being ±0.2 cm^−1^. The samples were treated in a potassium bromide matrix according to the literature data [27,28,29,30], after which the measurements were performed.

On a DD2 400 spectrometer (Agilent, Santa Clara, USA), the ^1^H NMR and ^13^C NMR IL spectra were recorded. Chemical shifts (δ) were defined in ppm for the compound solution in DMSO-d_6_, with the residual solvent peak as an internal standard. By comparing chemical shifts and peak multiplicities, assignments were proceeded and *J* values (in Hz) and carbon signals were clarified by HSQC experiments. Due to the NMR spectra of [mim][doc] and [bmim][doc] having been reported in our previous work [26], here we present the data for [empyrr][doc] and [hpyr] [doc].

[empyrr][doc]

^1^H NMR (400 MHz, DMSO-d_6_): δ 3.95–3.82 (m, 4H, OCH_2_), 3.64 (dd, *J* = 11.5, 3.7 Hz, 1H, CHSO_3_^−^), 3.54–3.35 (m, 6H, NCH_2_), 2.97 (s, 3H, NCH_3_), 2.91 (dd, *J* = 17.2, 11.5 Hz, 1H, CH_2_CO_2_), 2.79 (dd, *J* = 17.2, 3.7 Hz, 1H, CH_2_CO_2_), 2.14–2.00 (m, 4H, *CH_2_*CH_2_N), 1.53–1.44 (m, 2H, CH), 1.39–1.18 (m, 19H, CH_2_, *CH_3_*CH_2_N), and 0.91–0.77 (m, 12H, CH_3_).

^13^C NMR (101 MHz, DMSO-d_6_): δ 170.99 (CO_2_), 168.35 (CO_2_), 66.17 (OCH_2_), 66.10 (OCH_2_), 66.06 (OCH_2_), 66.02 (OCH_2_), 62.87 (NCH_2_), 62.84 (NCH_2_), 62.81 (NCH_2_), 61.41 (CSO_3_^−^), 58.32 (NCH_2_), 58.29(NCH_2_), 58.26(NCH_2_), 46.89 (NCH_3_), 46.85 (NCH_3_), 46.81 (NCH_3_), 38.18 (CH), 38.13 (CH), 38.10 (CH), 34.08 (CH_2_CO_2_), 29.73 (CH_2_), 29.62 (CH_2_), 29.56 (CH_2_), 28.35 (CH_2_), 28.33 (CH_2_), 23.18 (CH_2_), 23.16 (CH_2_), 23.00 (CH_2_), 22.98 (CH_2_), 22.41 (CH_2_), 22.38 (CH_2_), 21.06 (CH_2_), 13.88 (CH_3_), 13.85 (CH_3_), 10.78 (CH_3_), 10.75 (CH_3_), 10.71 (CH_3_), and 8.85 (NCH_2_*CH_3_*).

[hpyr] [doc]

^1^H NMR (400 MHz, DMSO-d_6_): δ 9.19 (d, *J* = 5.6 Hz, 2H, o-H-Ar), 8.63 (t, *J* = 7.8 Hz, 1H, *p*-H-Ar), 8.17 (dd, *J* = 7.4, 6.8 Hz, 2H, m-H-Ar), 4.65 (t, *J* = 7.5 Hz, 2H, NCH*_2_*), 3.95–3.80 (m, 4H, OCH_2_), 3.71 (dd, *J* = 11.5, 3.7 Hz, 1H, CHSO_3_^-^), 2.95 (dd, *J* = 17.2, 11.5 Hz, 1H, CH_2_CO_2_), 2.83 (dd, *J* = 17.2, 3.7 Hz, 1H, CH_2_CO_2_), 2.00–1.82 (m, 2H, NCH*_2_CH_2_*), 1.54–1.40 (m, 2H, CH), 1.39–1.15 (m, 22H, CH_2_), and 0.92–0.70 (m, 15H, CH_3_).

^13^C NMR (101 MHz, DMSO-d_6_): δ 170.93 (CO_2_), 168.40 (CO_2_), 145.50 (*p*-C-Ar), 144.82 (*o*-C-Ar), 128.10 (*m*-C-Ar), 66.20 (OCH_2_), 66.13 (OCH_2_), 66.04 (OCH_2_), 66.00 (OCH_2_), 61.45 (NCH_2_), 60.65 (CSO_3_^−^), 38.19 (CH), 38.16 (CH), 38.14 (CH), 38.13 (CH), 34.02 (CH_2_CO_2_), 30.81 (NCH_2_*CH_2_*), 29.75 (CH_2_), 29.73 (CH_2_), 29.63 (CH_2_), 29.56 (CH_2_), 28.35 (CH_2_), 25.05 (CH_2_), 23.18 (CH_2_), 23.15 (CH_2_), 23.00 (CH_2_), 22.41 (CH_2_), 22.39 (CH_2_), 21.86 (CH_2_), 13.81 (CH_3_), 13.78 (CH_3_), 13.71 (CH_3_), 10.71 (CH_3_), 10.68 (CH_3_), and 10.64 (CH_3_).

### 2.3. Density and Viscosity Measurements

A Stabinger Viscometer SVM 3000 (Anton Paar, Graz, Austria) with an error of 0.00005 g cm^−3^, was used to measure density and viscosity. The apparatus was calibrated by applying water at 298.15 K, after which the IL densities were measured at the same temperature. The received values were correlated with the literature data, with an average absolute deviation of 0.06%. Further measurements were carried out at temperatures of 293 K, 303 K, 313 K, 323 K, and 333 K at constant atmospheric pressure, with each measure repeated three times to assure reproducibility.

### 2.4. Differential Scanning Calorimetry

For differential scanning calorimetry (DSC) measurements, the following methodology was applied. Samples were located in open alumina pans and experiments were spent applying a thermal analyzer STA 449 F1 Jupiter (NETSCH, Selb, Germany). The thermograms were recorded in the range of −100–20 °C and had a ramp speed of 10 °C/min in an argon atmosphere.

### 2.5. Determination of Gases’ Solubility

**Figure 1:**
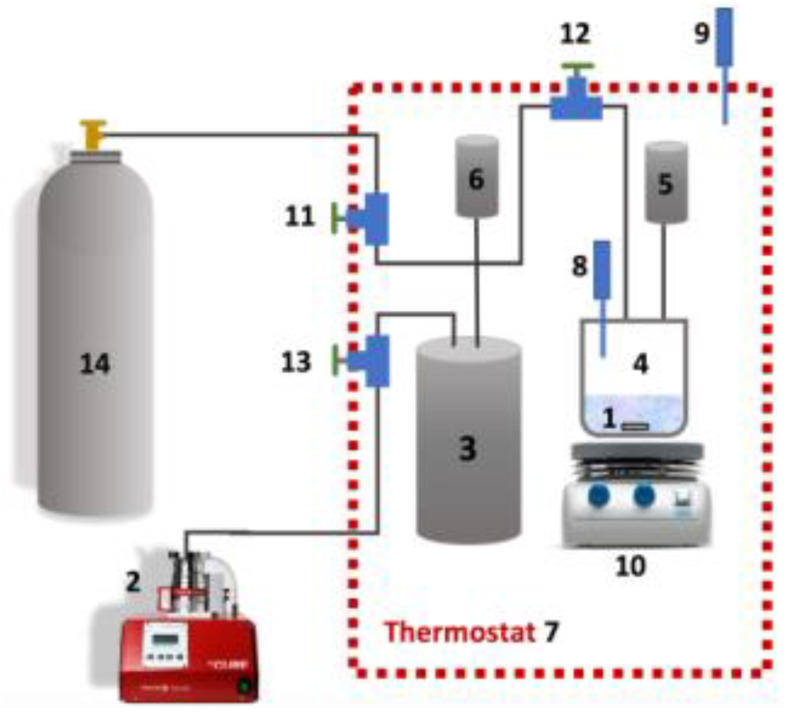
Scheme of the experimental setup: 1—sample; 2—vacuum pump; 3—gas container; 4—high-pressure equilibrium cell; 6—pressure sensor; 7—thermostatic air bath; 8, 9—temperature sensor; 10—magnetic stirrer; 11—13—valves; 14—gas cylinder.

The experimental setup is based on the pressure drop method, which is applied in most experimental investigations in the literature and is depicted in several publications. [31,32,33,34]. The installation scheme is presented in Figure 1. The setup consists of a high-pressure equilibrium cell (HPEC) (4) assured with a magnetic bar 16.72 cm^3^ in volume and a gas vessel (GC) (3) to add a certain amount of 35.24 cm^3^ CO_2_. A calibrated bulb of known volume applies to pre-calibrate and measure the volumes of the various compartments of the setup. The pressure in the GC and HPEC was controlled by applying two pressure sensors: CPG 1000 (5, 6) (WIKA GmbH, Germany) with a certainty ±0.05% for FS positive pressure and ±0.25% for FS vacuum/500 psi and below. The thermostatic air bath (7) with a temperature sensor (8, 9) (an accuracy of ±0.1 K) maintained a constant temperature. The pre-weighed quantity of ILs (1–2) was injected into the equilibrium cell and the system was evacuated with open valves (11) and (13). During the 24 h, the ILs were degassed and dehydrated in a vacuum at 343.15 K. Later, the valves were locked and a definite amount of gas (H_2_S or CO_2_) was injected into the GC (3) through the valve (11) from a cylinder with pure gas (H_2_S or CO_2_). After that, the valve on the HPEC (12) was opened so that the ILs could contact the gas. Determination of solubility at various temperatures was performed via the air thermostat.

Thus, this research makes it possible to carry out measurements in a wide temperature range (from 303.15 to 333.15 K) with a single loading of the setup. For the onset of absorption equilibrium, the constancy of the HPEC pressure for over 2 h was taken. Later, the difference between the two PVT measurements was applied to determine the amount of solute present in the ILs [2,35].

### 2.6. Computational Methods

A conductor-like screening model for real solvents, COSMO-RS is a new method for the prediction of thermophysical and chemical properties of fluids and liquid mixtures based on unimolecular quantum chemical calculations [13]. The full description of the methodology can be found elsewhere, and only major features needed for understanding the analysis and the discussion of the obtained results are highlighted here. Each COSMO-RS calculation includes two steps. The first step is the quantum chemical COSMO/DFT calculations of the chosen molecules. During this stage, each entity is individually inserted in a perfect conductor where a cavity is constructed around the molecule, so it induces a charge distribution in the discrete surface (the cavity) between the counterion and the conductor. These charges are called screening charges, and the surface density usually represents them. This charge distribution is considered the most important molecular descriptor. The obtained screening charge density of individual molecules is converted into the probability distribution function (histogram) p’(σ) or σ -profile. The σ -profile of a mixture p(σ) is built by adding the p0(σ) of the already screened molecules weighted by their mole fractions in the mixture. Having the σ-profile, the chemical potential is calculated by solving couples of non-linear equations. Finally, the obtained chemical potential is used to calculate thermodynamical properties such as activity coefficients, Henry’s law constants, solubilities of liquids and gases in IL, etc.

Density functional theory (DFT) calculations were performed to determine the fractional free volume of the ILs. The structural optimizations and quantum calculations were carried out using the ADF (Amsterdam Density Functional) program. The geometry optimization, the ADF COSMO calculations, and the parameters were optimized using the ADF program with the preset “COSMO-RS compound”. The settings of this preset are the Becke Perdew exchange correlation function (GGA:BP), the scalar relativistic ZORA Hamiltonian, a TZP small core basis set, and an integration accuracy with good quality.

## 3. Results and Discussion

### 3.1. Thermophysical Properties

**Figure 2:**
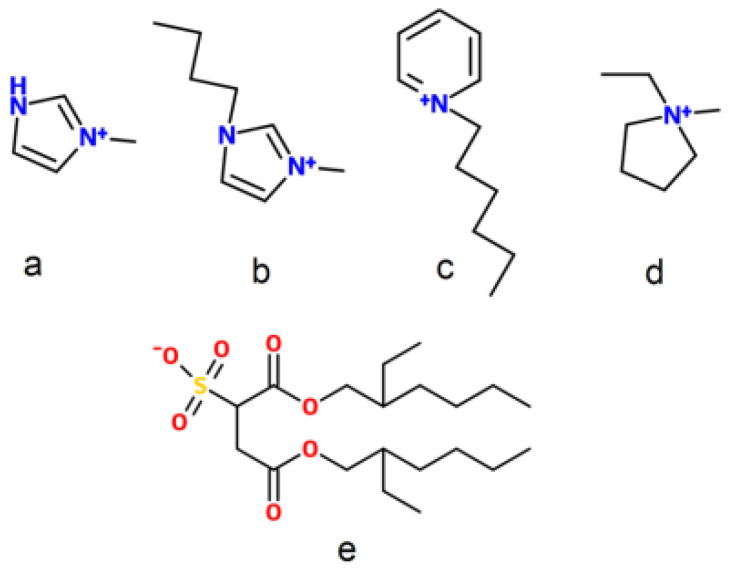
The structures of the cations and the [doc] anion. The abbreviations are as follows: (**a**) [mim], (**b**) [bmim], (**c**) [hpyr], (**d**) [empyrr], and (**e**) [doc].

The structures of the ILs based on amphiphilic bis(2-ethylhexyl) sulfosuccinate ([doc]) anion and 1-methylimidazoliun, 1 butyl-3-methylimidazolium, 1-hexylpyridinium, and 1-ethyl-1-methylpyrrolidinium cations are shown in Figure 2. NMR and FTIR spectroscopy were used to identify the structure of the ILs, which are represented in the Supplementary Materials (SM).

The thermal properties of [hpyr][doc] and [empyrr][doc] were studied using differential scanning calorimetry, which are also included in the SI and listed in Table 1. For [mim][doc] and [bmim][doc], the DSC results were reported in our previous work [9,36]. The melting points (T_m_) of the imidazolium-based ILs have varied in a range from 254 K to 260 K, while the pyridinium and pyrrolidinium salts do not have a melting point (T_m_). The latter two ILs were characterized by the glass transition temperature of approximately 223K.

**Figure 3:**
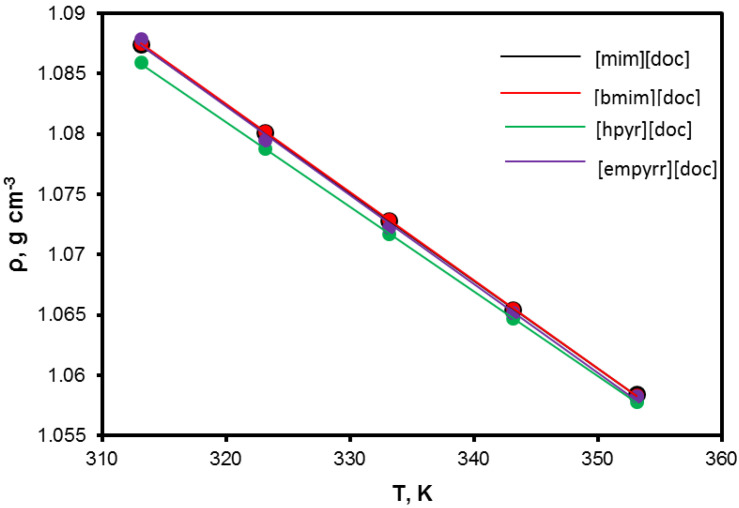
Density of the ionic liquids as a function of temperature.

The density is very important for technological design, which is a basic property for the characterization of any fluids. Moreover, a range of properties, such as thermal expansion or compressibility coefficients, are derived from density and provide information about the liquid structure and interactions. The density of investigated ILs, $\rho \left(T\right)$, experimentally obtained in a wide temperature range, are collected in Table S3 and plotted in Figure 3 as solid symbols. Due to the linear decreasing of the densities of all ILs with temperature within the whole measured temperature range, *T*, for fitting the obtained data a linear correlation (1), was used

(1) $$\rho =a_{0}+a_{1}\times T$$

The values of the coefficients $a_{0}$ and $a_{1}$, as well as their standard deviations, are represented in Table 1.

As depicted in Figure 3, whole ILs have shown minor differences in density. It means, basically, that all cations have comparable strength of interaction with the [doc] anion and the density value is defined more by the anion, while the cation’s contribution is not significant. For comparison, the same tendency was observed for ILs with a dicyanamide (DCA) anion with different cations, in which densities have very close values [37]. Additionally, it should be mentioned that the density values of obtained ILs are relatively low in comparison with known ILs. For example, at 313.15 K for ILs with [bmim] cation, the viscosity of [bmim][BF_4_], [bmim][PF_6_], [bmim][MeSO_4_], and [bmim][CF_3_SO_3_] are 1.1908, 1.3528, 1.1962, and 1.2924 g·cm^−3^, respectively, which is 11–26% higher than the density of [bmim][doc] (1.0729 g·cm^−3^) [38]. This fact should be attributed to the branched structure of (mainly) anion and sterically hinders the attraction between IL moieties, which cause the decrease in the strength of interactions and thus decrease the density [39]. As expected, the density values decrease almost linearly with increasing temperature. This linear behavior is common to ionic liquids in general and is a consequence of the large temperature difference between their working temperature range and their (inaccessible and therefore hypothetical) critical temperatures [40,41,42].

Compared with conventional ILs, the viscosities of the ILs synthesized have exhibited relatively higher values due to the bulky anion structure. From a practical sense, viscosity is a key issue affecting the permeability of supported ionic liquid membranes. Due to an inverse relationship between the viscosity and the diffusivity, for higher permeability of the gas separation membranes, it is preferable to diminish the viscosity of the ILs. However, in the case of [doc]-based SILMs, the low value of diffusion is compensated by the high solubility of particular gases (acidic gases). Interestingly, the unusual temperature effect was observed for the aforementioned SILMs. Unlike the conventional low viscous IL membranes, which are characterized by a decrease in selectivity with temperature, the selectivity of the membranes impregnated with [doc]-based IL remained almost unchanged. This phenomenon was caused by the decrease in the gas solubility is compensated by the intensification of diffusion, and selectivity undergoes insignificant changes.

**Figure 4:**
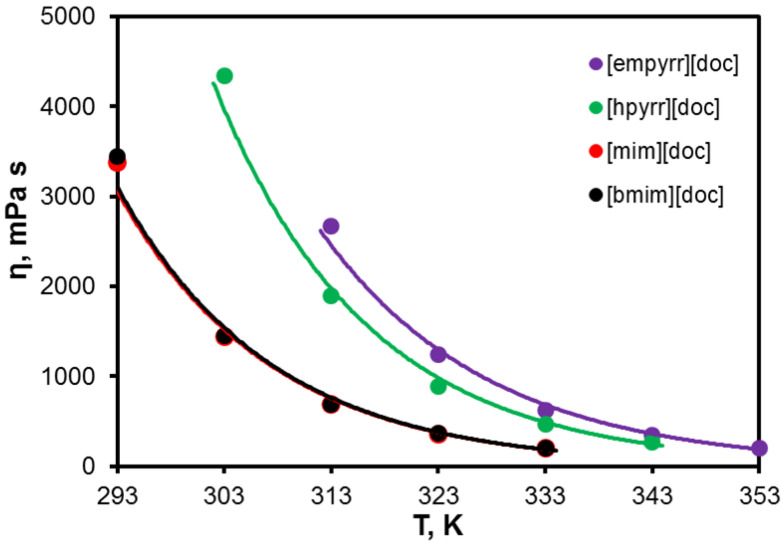
Viscosity of the ionic liquids as a function of temperature.

The experimental viscosity data (Figure 4, Table 2) were analyzed in order to determine the activation energy and to be fitted as a function of temperature, using the Guzman–Andrade equation [43,44]:

(2) $$\eta =\eta_{0}e^{E/RT}$$

where *η* is viscosity, mPa·s; *E* is the activation energy of viscous flow, kJ mol^−1^; *T* is the temperature, K; *R* is the the universal gas law constant, J·K^−1^ mol^−1^; and $\eta_{0}$ is viscosity at infinite temperature, mPa·s.

The activation energy for viscous flow E and the viscosity at infinite temperature η_0_ were determined from the plot slope (Table 3). The activation energy shows the energy required to overcome the intermolecular interactions between the molecules to change their positions. Higher values of activation energy match the stronger attraction of ion pairs, which is due to strong intermolecular interactions or in the attendance of steric hindrances. In general, the viscosities of the [doc]-based ILs varied in a range of 55–60 kJ/mol, depending on the steric hindrance of the cation.

### 3.2. Gas Sorption Properties

The SM consolidated data on the results of the solubility measurements of CO_2_ and H_2_S for temperatures of 303.2, 313.2, 323.2, and 333.2 K and pressures up to 12.7 bar. The precision and reliability of the measurement method were confirmed by dint of [bmim][Tf_2_N] [33,45]. The average reproducibility of the solubility data was within ±1%. The values of $H_{21.m}^{\left(0\right)}\;$ for each temperature T can be received from the points at the interception of Krichevsky–Kasarnovsky plots [46]. Henry’s law constants were obtained by extrapolating the available data on gas solubility in IL:

(3) $$H\;\left(T\right)=\underset{P\to 0}{\lim}\left[\frac{f_{i}\left(T,P\right)}{m_{i}/m^{0}}\right]$$

where *i* is the index number, indicating CO_2_ or H_2_S, *f*_i_(*T,P*) is the fugacity of *i* gas in the gas phase, $m_{CO_{2}}$ is the solubility of *i* gas in IL (on molality scale), and *m^o^* is the reference solubility (1 mol/kg).

Further, the changes in Gibbs free energy and entropy of H_2_S and CO_2_ absorption in the ILs at different temperatures can be calculated using the equations presented below. Thermodynamic solution properties (of CO_2_ in absorbents) can be determined from the above ratio of Henry constants using well-known thermodynamic relations [47]:

(4) $$\Delta_{sol}G=RT\ln\left(H_{21,m}\left(T,p\right)/p{}^{\circ}\right)$$

(5) $$\Delta_{sol}H=R{\left(\frac{\partial \ln\left(H_{21,m}\left(T,p\right)/p{}^{\circ}\right)}{\partial \left(1/T\right)}\right)}_{p}$$

(6) $$\Delta_{sol}S=\left(\Delta_{sol}H-\Delta_{sol}G\right)/T$$

where $\Delta_{sol}G$, $\Delta_{sol}H$ and $\Delta_{sol}S$ are the Gibbs free energy of solvation, enthalpy, and entropy of solvation, accordingly. The CO_2_ and H_2_S thermodynamic properties in the investigating absorbents were determined at standard temperature and pressure (T° = 298.15 K, p° = 1 bar). The $\Delta_{sol}H$ and $\Delta_{sol}S$ has negative values at any temperature for all absorbents. Moreover, an increase in temperature leads to a decrease in the values of these thermodynamic parameters. It has been established that the [bmim][doc] + H_2_S and [bmim][doc] + CO_2_ systems have similar $\Delta_{sol}S$ values (−44.14 and −42.39 J·mol^−1^·K^−1^), although the absorption of H_2_S was slightly higher than for CO_2_ ($H_{21.m}^{\left(0\right)}$= 2.91 vs. 9.79 bar at 303.2 K). Since more negative $\Delta_{sol}H$ value results from lower $\Delta_{sol}G$ in the absorption, the dissolution in the [doc]-based IL will be thermodynamically more favorable for H_2_S than for CO_2_. Thermodynamic properties of CO_2_ and H_2_S in the ILs can be seen in Table 4.

H_2_S showed relatively high solubility values in the studied ILs, while the Henry constant for H_2_S at 303.2 K in absorbents reached 2.91 and 8.31 bar, accordingly. The [mim][doc] showed a much lower solubility value than other samplers; the best value for [mim][doc] was 30.03 bar for CO_2_. The results obtained are most likely due to the protonic nature of [mim][doc] having a weakly acidic protonated nitrogen in the imidazolium cation. Correspondingly, the cooperation of a proton with an alkaline anion will decrease the proximity of the IL to the acid gases. The minimal H_2_S solubility was found for [empyrr][doc]. In the case of H_2_S, we should take into account the cumulative effect of the free volume, hydrogen bonding, and protic nature of the cation on the solubility. The IL-containing [empyrr] cation had slightly higher solubilities than the [hpyr]-based one, probably, due to the differences in free volume. Thus, among the studied absorbents, the [mim][doc] had the lowest solubility value, whereas the [bmim][doc] had the highest solubility value.

COSMO-RS was used to calculate IL-free volume and solubility values. Although the small difference in the IL density indicates an approximately equal absorbent free volume, the free volume effect may explain the observed variations of sorption capacity.

**Figure 5:**
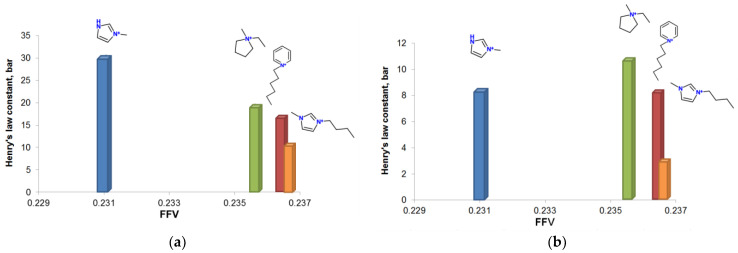
Henry’s law constants of CO_2_ (**a**) and H_2_S (**b**) as a function of the fractional free volume for [doc]-based ionic liquids.

The volumetric effects arising from the cation changes are analyzed using the data illustrated in Figure 5. The fraction-free volumes (FFV) available in the ILs are calculated according to the procedure proposed by Shannon et al., [48] as follows:

(7) $$FFV=\frac{V_{m}-1.3V_{vdw}}{V_{m}}\approx \frac{V_{m}-V_{COSMO}}{V_{m}}$$

where V_m_ is the molar volume of the IL in cm^3^/mol; V_vdw_ is its van der Waals volume of the IL molecule in cm^3^/mol; and V_COSMO_ is the “COSMO volume” of the IL, which is the volume enclosed by the COSMO surface in cm^3^/mol. The molar volume can be determined from the experimental density:

(8) V_m_ = M_r_/ρ

where M_r_ is the relative molar mass of the IL.

Indeed, through the analysis of Henry’s law constants of the ILs depending on the FFV, we have observed an antibate (reciprocal) correlation between those two parameters. Increasing the FFV in a range of the cations [mim] < [empyrr] < [hpyr] < [bmim] resulted in a decrease in the sorption capacity of CO_2_. As mentioned before, the drastic change of Henry’s law constant in the case of [mim] is explained in terms of the acidic nature of the cation, while diminishing the FFV in other cations leads to a minor increase in sorption properties. We have observed a similar tendency in the case of hydrogen sulfide sorption, excepting Henry’s law constant in [mim][doc].

Most probably, the presence of acidic hydrogen in the C(2) of the imidazolium heterocycle has led to the higher solubility of H_2_S than in the ILs with other heterocyclic cations due to the hydrogen bonding. A comparison of literature data and experimental results presented in Table 5 was performed to determine the absorption capacity for conventional and synthesized ILs. Generally, acid gas solubility for synthesized ILs was higher in comparison with conventional ILs. For example, the CO_2_ solubility in [bmim][doc] is almost 4–5 times higher than in conventional ILs. For H_2_S, the solubility indices were approximately 2.5–6 times higher for the synthesized ILs.

**Figure 6:**
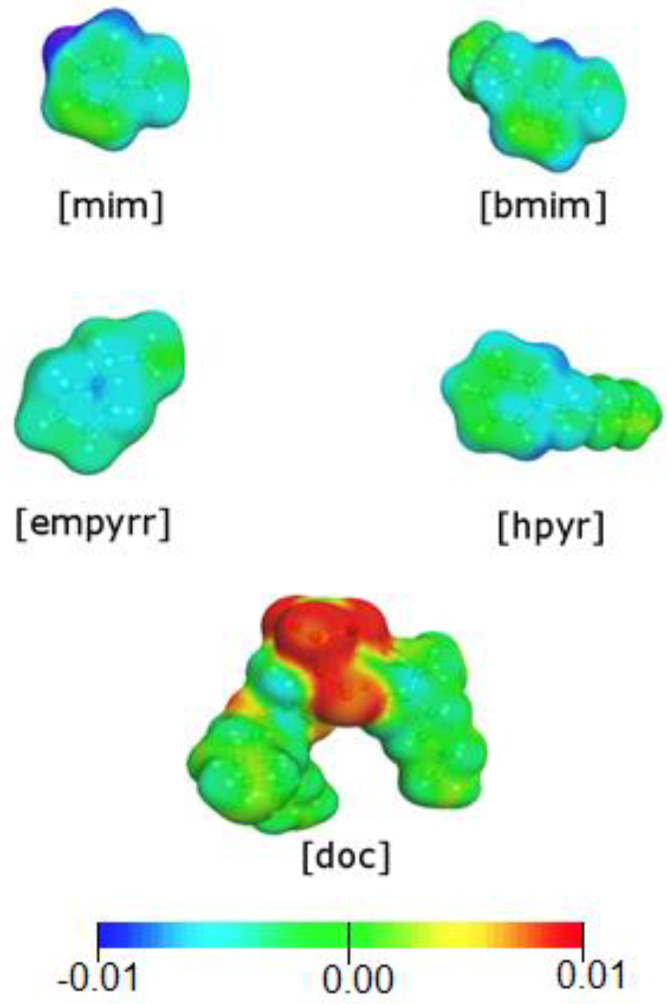
COSMO surface charge distribution for [doc]-based ionic liquids.

To visualize the structural variations in cations depending on the solubility of gases of the studied ILs, DFT calculations were performed. For different heterocycles, the charge distribution on the surface of cations will have varies considerably (Figure 6). Two acidic protons N(3)-H and C(2)-H were observed in the structure of [mim] cation and N(3)-H proton acidity was more pronounced. In the case of [bmim] cation, the most acidic site was placed in the C(2)-H bond. The positive charge in the [empyrr] cation was localized in the nitrogen atom. However, due to the presence of alkyl substituents at the nitrogen atom on the COSMO surface, the charge turned out to be equally distributed. The acidity of C(2)-H and C(6)-H protons turned out to be higher than other protons in the [hpyr] heterocycle.

## 4. Conclusions

The calculation results showed that the CO_2_ solubility in all prepared ILs is created by van der Waals forces and ranges from 12.01 to 12.14 kJ/mol, while the energy of electrostatic interactions, both for CO_2_ and H_2_S, plays a secondary role for their sorption in four ionic liquids and has a slight cation dependence, and is about 4.27–4.48 kJ l/mol for CO_2_ and 4.68–4.73 kJ/mol for H_2_S, respectively. Another type of interaction affects the H_2_S solubility, such as hydrogen bonds, and the interaction energy is from 2.89 to 3.18 kJ/mol. The calculation results showed the highest affinity of the ionic liquid, and the dissolved gas was found for the ionic liquid based on the cation [bmim] is also comparable with the experimental results obtained.

We have examined the sorption capacity of ionic liquids containing a nonfluorinated bulky anion bis(2-ethylhexyl) sulfosuccinate and four cations, namely N-methylimidazolium [mim], 1-butyl-3-methylimidazolium [bmim], 1-ethyl-1-methylpyrrolidinium bromide [empyrr], and 1-hexylpyridinium [hpyr]. The experimental results of H_2_S and CO_2_ solubility indicate that these ionic liquids are able to dissolve a relatively high amount of gas via the physical absorption mechanism. It was observed that the protic nature of the cation leads to a remarkable reduction in acidic gas solubility, while the structure of aprotic cations has slightly affected the sorption capacity of the ILs.

To understand the influence of the cation, the fractional free volumes of the four ILs were calculated and considered together with Henry’s law constants. As expected, the lowest fractional free volume values corresponded to the highest Henry’s constants. The solubility data correlated with the free volume calculations were ranked as follows: [mim] < [empyrr] < [hpyr] < [bmim]. The bmim [doc] was characterized by higher absorption capacity toward the acidic gases than other ILs. In addition, negligible distinctions in density and activation energies of the viscous flow were found for the four tested ILs. Thus, the concept based on the insertion of sterically hindered moieties along with polar functional groups in the anion structure is applicable to the development of H_2_S and CO_2_-selective absorbents.

## Figures and Tables

**Table 1:** Obtained by fitting the experimental data on density with eq. 1 coefficients $a_{0}$ and $a_{1}$, the standard deviations of the obtained parameters, the fit standard errors, $\sigma_{\mathrm{fit}}$, and *r*^2^ of the corresponding correlations.

Parameters	[mim][doc]	[bmim][doc]	[hpyr][doc]	[empyrr][doc]
$a_{0}$/g·cm^−3^	1.301 ± 0.001	1.3018 ± 0.0005	1.318 ± 0.005	1.2990 ± 0.0008
$10^{4}\cdot a_{1}/$g·cm^−3^·K^−1^	–7.27 ± 0.04	–7.31 ± 0.02	–7.4 ± 0.2	–7.03 ± 0.03
$10^{4}\cdot \sigma_{\mathrm{fit}}$/g·cm^−3^	1.14	0.48	5.16	0.80
*r* ^2^	0.99985	0.99997	0.99705	0.99992

**Table 2:** Physical properties of the [doc]-based ILs.

Ionic Liquid	Molar Weight, g mol^−1^	Density, g cm^−3^	Molar Volume, cm^3^ mol^−1^	T_m_, K	Viscosity (313 K), mPa s	Water Content, ppm	Reference
[mim][doc]	505.12	1.0874	464.5208755	260	683.9	17.0	[9]
[bmim][doc]	561.2	1.0875	516.045977	254	692.3	25.6	[9]
[hpyr][doc]	583.56	1.0937	533.564963	-	1895.2	32.8	in this work
[empyrr][doc]	535.67	1.1125	481.5011236	-	2670.0	22.3	in this work

**Table 3:** The activation energy for ILs, calculated by the Guzman–Andrade equation.

Ionic Liquid	$\eta_{0}\cdot 10^{10},\;\mathrm{mPa}\cdot s$	E, kJ/mol	Correlation Coefficient, R^2^
[mim][doc]	2.00	56.87	0.9980
[bmim][doc]	2.00	57.03	0.9979
[hpyr][doc]	2.00	60.37	0.9983
[empyrr][doc]	4.00	58.78	0.9982

**Table 4:** Thermodynamic properties of CO_2_ and H_2_S in the ILs.

T/K	$H_{21.m}^{\left(0\right)}$/bar		$\Delta_{sol}G/\mathrm{kJ}\cdot {\mathrm{mol}}^{-1}$		$\Delta_{sol}H/\mathrm{kJ}\cdot {\mathrm{mol}}^{-1}$		$\Delta_{sol}S/J\cdot {\mathrm{mol}}^{-1}\cdot K^{-1}$	
**[bmim][doc]**								
	CO_2_	H_2_S	CO_2_	H_2_S	CO_2_	H_2_S	CO_2_	H_2_S
303.2	9.79	2.91	5.75	2.69	−7.63	−10.16	−44.14	−42.39
313.2	11.39	3.46	6.33	3.23	−8.68	−11.77	−47.94	−47.90
323.2	12.75	4.09	6.84	3.79	−9.82	−13.52	−51.54	−53.56
333.2	13.65	4.54	7.24	4.19	−11.05	−15.43	−54.87	−58.87
**[mim][doc]**								
303.2	30.03	8.31	8.57	5.33	−4.11	−4.12	−41.82	−31.17
313.2	31.74	8.82	9.00	5.67	−4.48	−4.72	−43.04	−33.15
323.2	33.68	9.44	9.45	6.03	−4.88	−5.37	−44.31	−35.28
333.2	35.61	9.96	9.89	6.37	−5.29	−6.08	−45.57	−37.35
**[empyrr][doc]**								
303.2	19.14	10.63	7.44	5.96	−10.08	−7.47	−57.76	−44.28
313.2	21.33	11.63	7.96	6.38	−11.35	−8.49	−61.68	−47.51
323.2	24.78	13.02	8.62	6.89	−12.73	−9.60	−66.07	−51.03
333.2	29.55	14.74	9.38	7.45	−14.22	−10.79	−70.80	−54.76
**[hpyr][doc]**								
303.2	16.58	8.21	7.08	5.30	−11.66	−7.39	−61.79	−41.88
313.2	19.66	9.71	7.75	5.92	−13.17	−8.44	−66.81	−45.85
323.2	23.22	10.35	8.45	6.28	−14.81	−9.58	−71.96	−49.07
333.2	27.44	11.55	9.17	6.77	−16.57	−10.81	−77.25	−52.78

**Table 5:** The CO_2_ and H_2_S Henry’s law constants.

	$H_{21.m}^{\left(0\right)}/\mathrm{bar}$				Ref.
	303.2 K	313.2 K	323.2 K	333.2 K	
CO_2_					
[bmim][doc]	9.8	11.39	12.75	13.65	in this work
[mim][doc]	30.03	31.74	33.68	35.61	in this work
[empyrr][doc]	19.14	21.33	24.78	29.55	in this work
[hpyr][doc]	16.58	19.66	23.22	27.44	in this work
[bmim][PF_6_]	59	-	81.3	-	[45]
[bmim][BF_4_]	60	68	75	81	[49]
[bmim][Tf_2_N]	42	45	51	-	[49]
H_2_S					
[bmim][doc]	2.91	3.46	4.09	4.54	in this work
[mim][doc]	8.31	8.82	9.44	9.96	in this work
[empyrr][doc]	10.63	11.63	13.02	14.74	in this work
[hpyr][doc]	8.21	9.71	10.35	11.55	in this work
[bmim][PF_6_]	18.6	21.6	25.7	30.9	[45]
[bmim][BF_4_]	15.5	19.1	23.4	28.5	[49]
[bmim][Tf_2_N]	13.7	16.5	18.9	21.7	[49]

## Data Availability

Not applicable.

## Supplementary Materials

The following are available online at https://www.mdpi.com/article/10.3390/membranes13020238/s1. Supporting Information includes the results of IR, NMR, DSC, the density and viscosity, CO_2_, and H_2_S solubility measurements at temperatures of 303.2, 313.2, 323.2, and 333.2 K and pressures of up to 12.7 bar.
