# Supplementary material for: The Cation Effect on the Free Volume and the Solubility of H2S and CO2 in Ionic Liquids Based on Bis(2-Ethylhexyl) Sulfosuccinate Anion
Source: Membranes (Basel). 2023 Feb 16;13(2):238. doi: 10.3390/membranes13020238 (PMC9965390; doi:10.3390/membranes13020238)
Supplement: Supplementary file 1 [file membranes-13-00238-s001.zip › membranes-2180273-supplementary.pdf]

membranes

MDPI

Supplementary material

The Cation Effect on the Free Volume and the Solubility of H_2S and CO_2 in Ionic Liquids Based on Bis(2-Ethylhexyl) Sulfosuccinate Anion

Tatyana S. Sazanova [1], Alsu I. Akhmetshina [2,†], Anton N. Petukhov [1,3,*], Andrey V. Vorotyntsev [3], Sergey S. Suvorov [3], Alexandra V. Barysheva [3], Amal Mechergui [1], Alexander V. Nyuchev [3], Olga V. Kazarina [1,3], Anna N. Stepakova [1], Maria E. Atlaskina [1], Artem A. Atlaskin [1], Sergey S. Kryuchkov [1] and Ilya V. Vorotyntsev [1]

1 Laboratory of SMART Polymeric Materials and Technologies, Mendeleev University of Chemical Technology, 9 Miusskaya Sq., Moscow 125047, Russia
2 Department of Technologies for the processing of polymers and composite materials, Kazan National Research Technological University, 68 Karl Marx Str., Kazan 420015, Russia
3 Chemical Engineering Laboratory, Research Institute for Chemistry, N.I. Lobachevsky State University of Nizhny Novgorod, 23 Gagarin Avenue, Nizhny Novgorod 603950, Russia
* Correspondence: antopetukhov@gmail.com
† Deceased author.

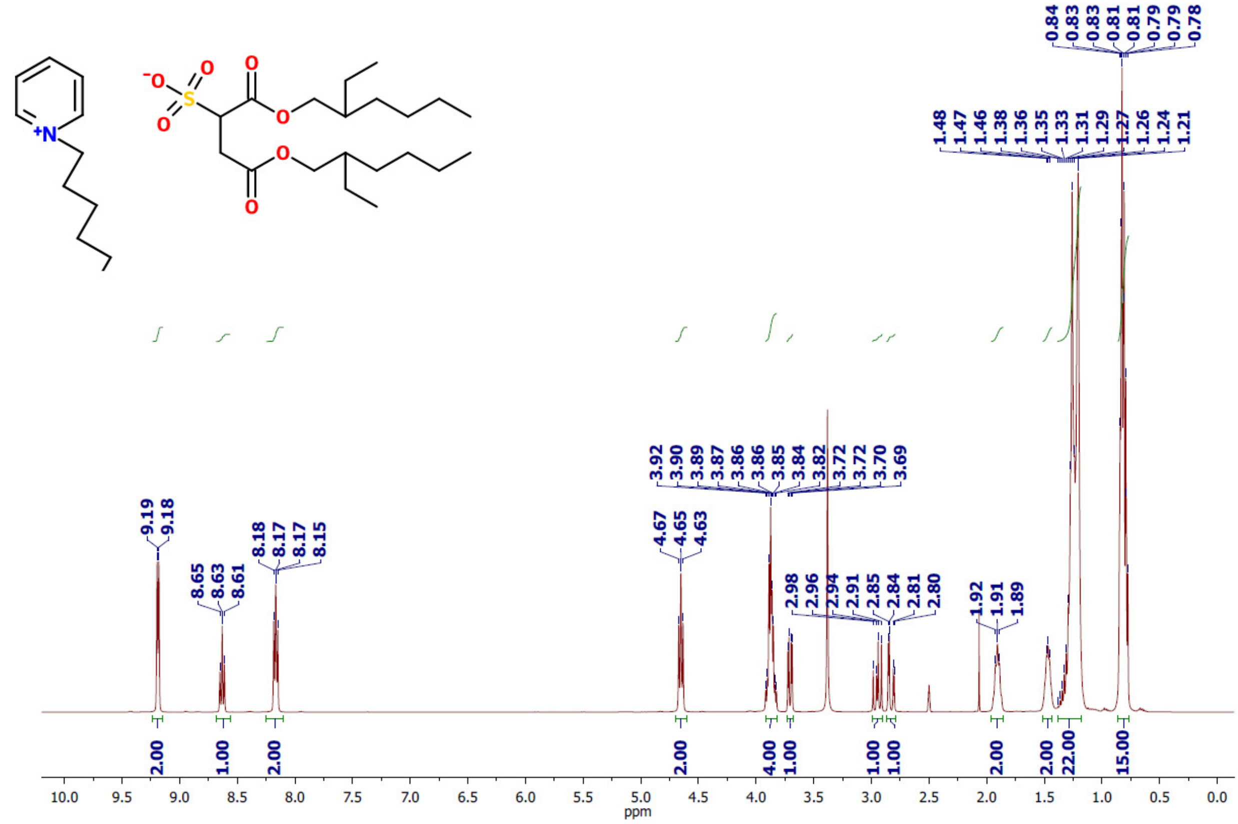

A

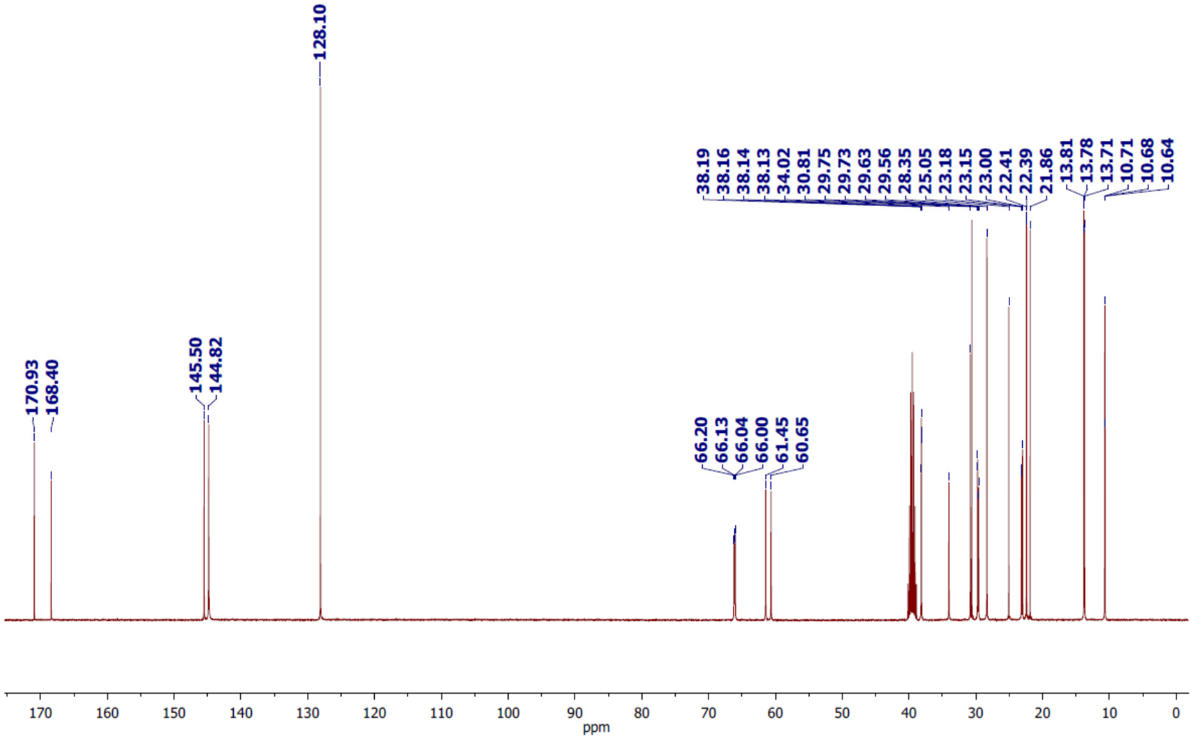

B

Figure S1. The ¹H (a) and ¹³C NMR (b) of 1- hexylpyridinium bis(2-ethylhexyl) sulfosuccinate.

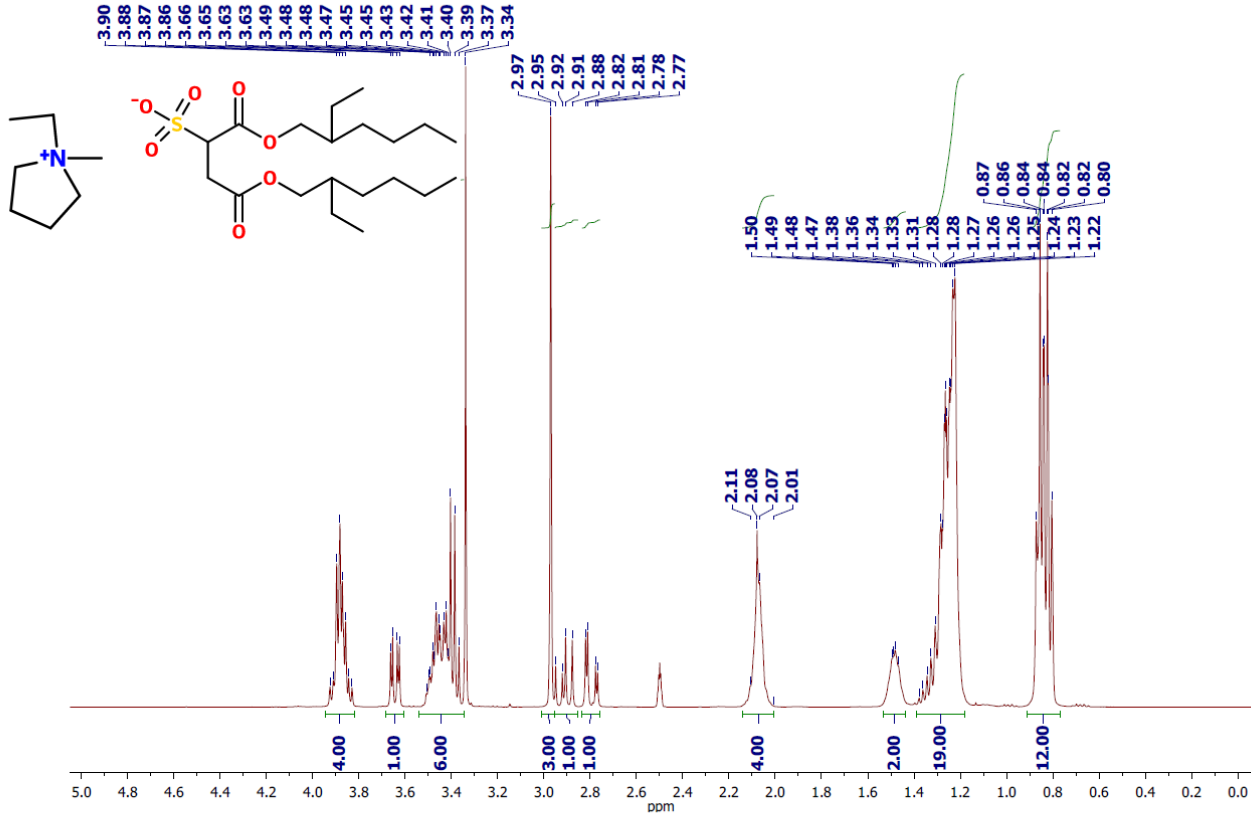

A

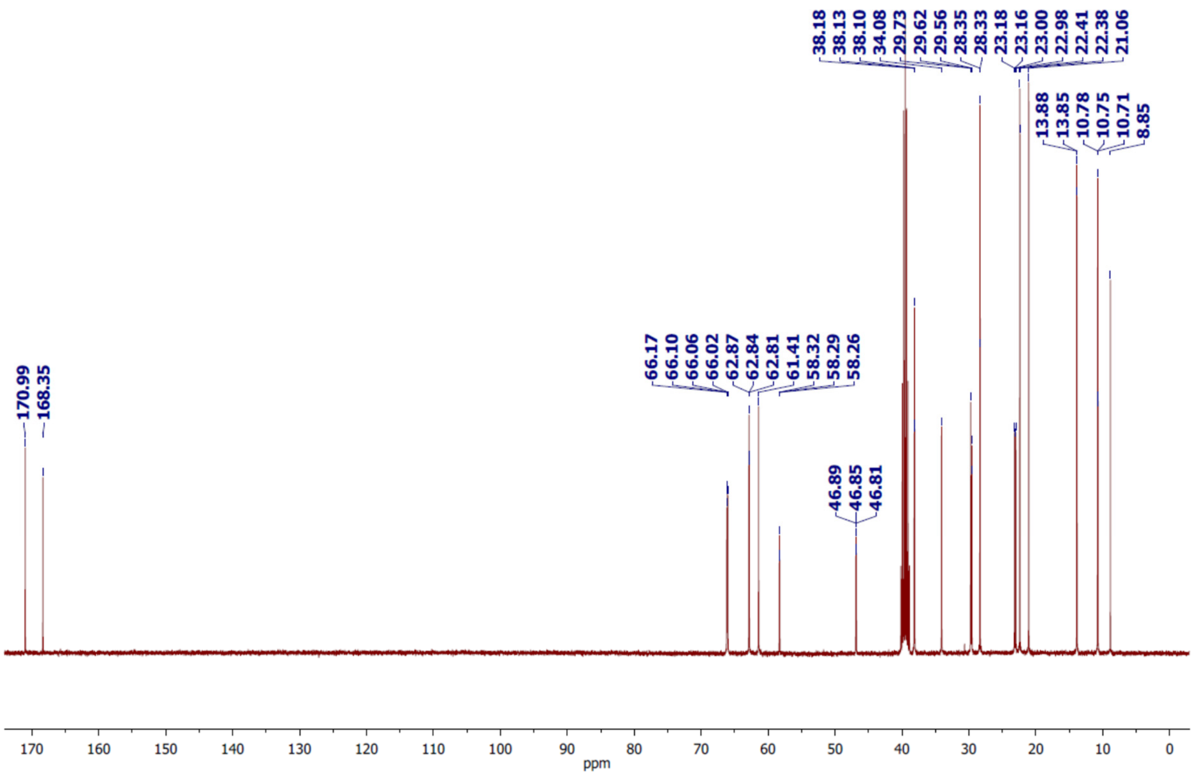

B

Figure S2. The ^{1}H (**a**) and ^{13}C NMR (**b**) of 1-ethyl-1-methylpyrrolidinium bis(2-ethylhexyl) sulfosuccinate.

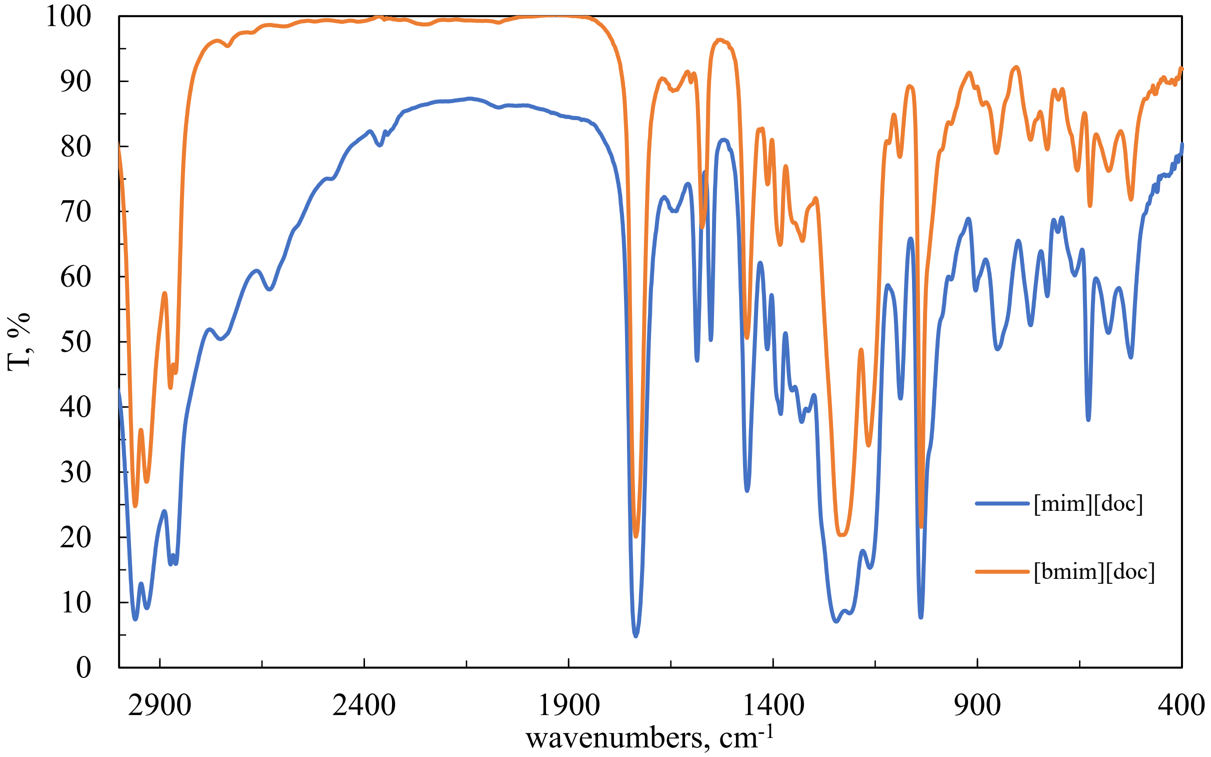

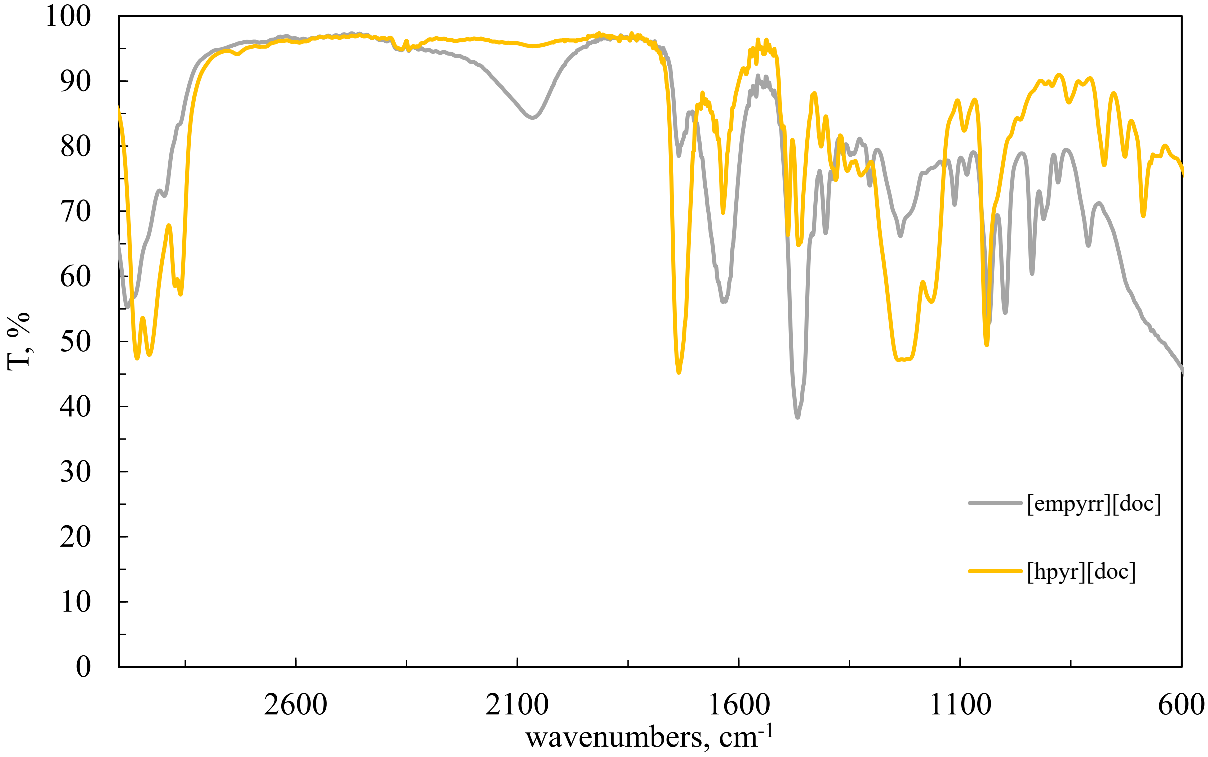

Figure S3. The FTIR spectra of the [doc] – based ILs.

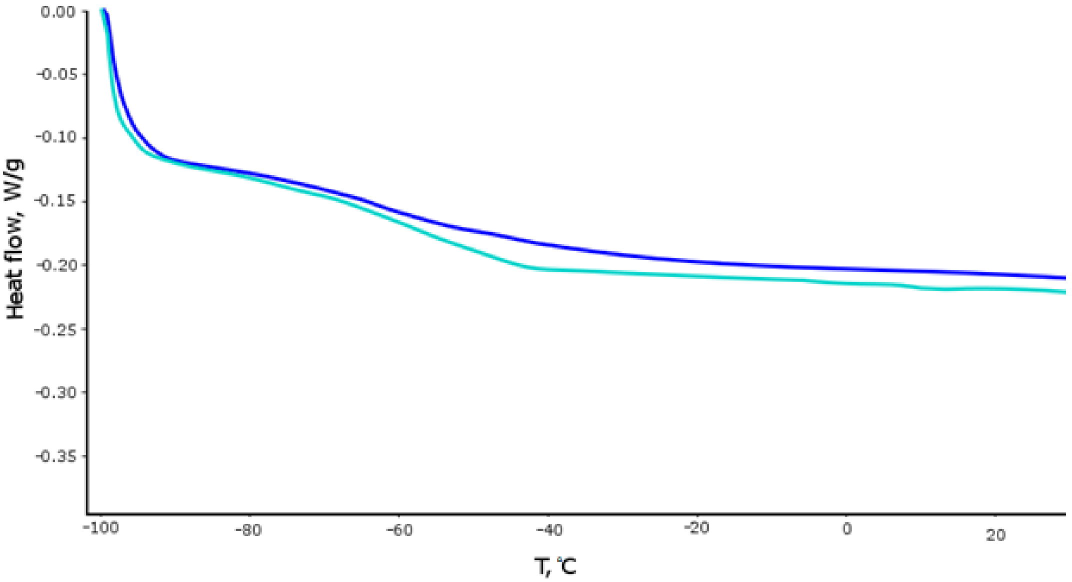

Figure S4. DSC thermograms of and 1-ethyl-1-methylpyrrolidinium bis(2-ethylhexyl) sulfosuccinate (**a**) 1- hexylpyridinium bis(2-ethylhexyl) sulfosuccinate (**b**).

Table S1. Mole Fraction Solubility of Carbon Dioxide Gas.

T/K	[bmim][doc]		[mim][doc]	
	P (bar)	m_{CO_2}/ $(mol_{CO_2}/\mathrm{kg_{IL}})$	P (bar)	m_{CO_2}/ $(mol_{CO_2}/\mathrm{kg_{IL}})$
303.2	2.210	0.207	2.073	0.067
	3.618	0.322	3.477	0.111
	5.152	0.433	4.878	0.153
	6.590	0.527	6.275	0.193
313.2	2.362	0.193	2.312	0.071
	3.928	0.301	3.654	0.110
	5.515	0.403	5.130	0.152
	6.877	0.490	6.411	0.187
323.2	2.516	0.186	2.652	0.077
	3.999	0.279	3.995	0.114
	5.589	0.377	5.487	0.155
	6.985	0.460	6.813	0.189
333.2	2.658	0.1818	2.925	0.080
	4.351	0.2773	4.283	0.115
	5.876	0.3626	5.750	0.153
	7.279	0.438	7.066	0.186
	[hpyr][doc]		[empyrr][doc]	
	P (bar)	m_{CO_2}/ $(mol_{CO_2}/\mathrm{kg_{IL}})$	P (bar)	m_{CO_2}/ $(mol_{CO_2}/\mathrm{kg_{IL}})$
303.2	2.191	0.095	2.088	0.103
	3.577	0.140	3.505	0.154
	4.662	0.169	5.016	0.197
	6.286	0.205	6.187	0.224
313.2	2.213	0.088	2.225	0.096
	3.672	0.132	3.671	0.142
	4.776	0.160	5.184	0.183
	6.392	0.195	6.383	0.210
323.2	2.230	0.078	2.239	0.084
	3.784	0.121	3.687	0.128
	4.934	0.148	5.258	0.168
	6.655	0.183	6.410	0.194
333.2	2.478	0.074	2.375	0.078
	3.994	0.110	3.797	0.117
	5.180	0.135	5.419	0.156
	6.904	0.167	6.588	0.181

Table S2. Mole Fraction Solubility of Hydrogen Sulfide Gas.

**T/K**	[bmim][doc]		[mim][doc]	
	P (bar)	m_{CO_2}/ $(mol_{CO_2}/\mathrm{kg_{IL}})$	P (bar)	m_{CO_2}/ $(mol_{CO_2}/\mathrm{kg_{IL}})$
303.2	2.010	0.722	1.995	0.247
	3.183	1.187	3.365	0.426
	4.277	1.657	4.280	0.550
	6.110	2.457	5.428	0.711
313.2	2.122	0.626	2.179	0.252
	3.343	1.038	3.529	0.414
	4.439	1.394	4.402	0.527
	6.301	2.055	5.606	0.674
323.2	2.355	0.591	2.293	0.249
	3.620	0.940	3.651	0.340
	4.690	1.252	4.527	0.505

	6.569	1.767	5.768	0.651
333.2	2.775	0.627	2.478	0.258
	4.009	0.935	3.807	0.401
	5.136	1.214	4.692	0.507
	6.970	1.666	5.945	0.652
	[hpyr][doc]		[empyrr][doc]	
	P (bar)	m_{H_2S}/ $(mol_{H_2S}/\mathrm{kg_{IL}})$	P (bar)	m_{H_2S}/ $(mol_{H_2S}/\mathrm{kg_{IL}})$
303.2	2.311	0.247	2.209	0.303
	3.545	0.410	3.451	0.512
	4.700	0.590	4.507	0.718
	5.789	0.789	6.848	1.312
313.2	2.449	0.241	2.291	0.267
	3.643	0.388	3.520	0.443
	4.776	0.550	4.645	0.631
	5.902	0.740	6.990	1.143
323.2	2.451	0.216	2.420	0.269
	3.714	0.355	3.621	0.436
	4.953	0.517	4.779	0.626
	6.201	0.713	7.216	1.166
333.2	2.580	0.201	2.491	0.250
	3.785	0.317	3.763	0.412
	5.008	0.455	4.962	0.595
	6.315	0.630	7.279	1.075

Table S3. Density and viscosity of the ILs as a function of temperature.

**T / K**	ϱ (g·cm⁻³)		η (mPa·s)	
	[mim][doc]	[bmim][doc]	[mim][doc]	[bmim][doc]
293.15	1.0874	1.0875	3381.1	3441.7
303.15	1.0801	1.0802	1439.9	1454
313.15	1.0728	1.0729	683.91	692.32
323.15	1.0654	1.0655	359.48	363.28
333.15	1.0584	1.0583	204.86	206.52
T / K	ϱ (g·cm⁻³)		η (mPa·s)	
	[empyrr][doc]	[hpyr][doc]	[empyrr][doc]	[hpyr][doc]
303.15	-	1.0859	-	4342.2
313.15	1.0879	1.0788	2670	1895.2
323.15	1.0795	1.0717	1242.3	893.16
333.15	1.07227	1.0647	628.36	468.13
343.15	1.0652	1.0578	346.75	267.58
353.15	1.05826	-	207.47	-
